# Clinical, Laboratory and Neurodevelopmental Findings in Children from the Yanomami-Ninam Population Chronically Exposed to Methylmercury

**DOI:** 10.3390/toxics12030193

**Published:** 2024-03-01

**Authors:** Adriana Duringer Jacques, Mirian Akiko Furutani de Oliveira, Mayara Calixto da Silva, Cristina Barroso Hofer, Paulo Cesar Basta

**Affiliations:** 1 Postgraduate Program, Epidemiology in Public Health, National School of Public Health, Oswaldo Cruz Foundation (EPSJV/Fiocruz), Av. Brasil, 4365-Manguinhos, Rio de Janeiro 21040-900, RJ, Brazil; mayaracx_2010@hotmail.com; 2 Psychology Division, Central Institute of the Hospital das Clínicas, School of Medicine, University of São Paulo (DIP/ICHC-FMUSP), São Paulo 05403-000, SP, Brazil; mirian.akiko@hc.fm.usp.br; 3 Department of Infectious Diseases, Federal University of Rio de Janeiro (UFRJ), Rio de Janeiro 21941-630, RJ, Brazil; cbhofer@hucff.ufrj.com.br; 4 Department of Endemics Diseases Samuel Pessoa, National School of Public Health, Oswaldo Cruz Foundation (ENSP/Fiocruz), Rua Leopoldo Bulhões, 1480-Manguinhos, Rio de Janeiro 21041-210, RJ, Brazil

**Keywords:** mercury exposure, methylmercury, indigenous people, indigenous children, neurodevelopment, Brazilian Amazon

## Abstract

Despite legal safeguards, the Yanomami community faces challenges such as unauthorized incursions by gold miners, resulting in environmental degradation, particularly from mercury. This jeopardizes the health and food security of indigenous individuals, especially due to the consumption of contaminated fish. Ethnic and racial disparities persist in indigenous healthcare, marked by troubling health indicators such as malnutrition, anemia, and infectious diseases. This cross-sectional study, conducted in October 2022 in the Yanomami Indigenous Territory in the Amazon Forest, Brazil, presented clinical, laboratory, and neurodevelopmental findings in Yanomami children chronically exposed to methylmercury. The results revealed that Yanomami children exhibited weights and heights below expectations (median Z-scores of −1.855 for weight for age and −2.7 for height for age), a high prevalence of anemia (25%), low vaccination coverage (15%), and low IQ (average 68.6). The Total Hair Mercury (Total Hg) levels ranged from 0.16 µg/g to 10.20 µg/g (mean: 3.30 µg/g; median: 3.70 µg/g). Of 117 children tested, 93 children (79.4%) had levels ≥ 2.0 µg/g (had no significant difference between sex). Among the 58 children for whom it was possible to estimate the Total Intelligence Quotient (TIQ), the average value was 68.6, ranging from 42 to 92 points (median: 69.5; standard deviation: 10.5). Additionally, the lowest score on the IQ test was associated with 5 times the risk of having high levels of mercury in their hair, 2,5 fold the risk of having an older age, and almost 8 times the risk of consuming fish, adjusting for nut consumption. Notwithstanding the study’s limitations, results suggest that mercury contamination from illegal mining activities on indigenous lands may negatively impact neurodevelopment in older indigenous children, particularly those fish consumers, despite the inherent benefits of fish consumption. Addressing other socio-environmental concerns is crucial for enhancing the overall health of the population.

## 1. Introduction

The Yanomami indigenous community has lived in the Amazon region for centuries, in territories shared between Brazil and Venezuela, in accordance with their traditions and customs. They practice subsistence agriculture, hunting, fishing, and gathering, maintaining an intimate relationship with nature and the tropical forest [1]. The Yanomami Indigenous Territory is a demarcated and protected area in Brazil that covers an extensive region in the state of Roraima and part of the state of Amazonas [2].

The Brazilian constitution of 1988 determined the recognition of the sociocultural and territorial rights of indigenous peoples, in addition to the creation of the Unified Health System (SUS, acronym in Portuguese), whose fundamental principle is universal and equal access to health for all Brazilian citizens, including indigenous peoples [3]. Subsequently, in 1999, the Indigenous Health Subsystem (Sasi-SUS, acronym in Portuguese) was created, which has the specific objective of meeting the health needs of indigenous populations according to their cultural and territorial characteristics. Despite legal advances and the creation of these health systems, ethnic-racial inequalities still exist in indigenous health care [4].

In spite of the recognition of territorial rights, the search for gold does not respect borders and, consequently, the invasion of prospectors into Yanomami indigenous lands, in addition to being an illegal practice, causes adverse impacts on the environment, such as deforestation, contamination of rivers by mercury, and soil degradation. These activities also have direct effects on biodiversity, vegetation, and the well-being of indigenous communities, compromising their health and generating food insecurity, increased violence, and scarcity of natural resources [2,5,6].

Mining operations result in the emission of inorganic mercury (Hg) into the ecosystem, which is subsequently subjected to biotransformation processes when it encounters aquatic environments, converting into methylmercury (MeHg). MeHg is absorbed by animals, and as they move up the food chain, its concentration increases. Populations that have a diet based on fish, such as the indigenous people of the Amazon, are particularly vulnerable to methylmercury contamination [7,8]. A study carried out between 1994 and 1995, in a section of the Yanomami Territory affected by mining activity, found mercury levels in fish and indigenous people that exceeded the limits established by the World Health Organization (WHO) as safe [9]. The growth of illegal mining in the Yanomami Indigenous Territory therefore leads to a series of health concerns such as the high prevalence of malaria, cases of malnutrition, and respiratory diseases [5]. A reflection of this is the alarmingly high rate of preventable deaths in children under five years of age, which reached 2275 deaths per 100,000 inhabitants between 2019 and 2020, being 13.7 times higher compared to non-indigenous children of the same age in the Brazilian population [10].

Some studies have demonstrated the association between methylmercury exposure and intelligence in Amazonian children, especially by the exposure of the gold mining activities [11,12]. Some other authors have shown the association between methylmercury and other cognitive abilities, such as working memory, in indigenous population [13] and visuospatial skills in Amazonian children [14]. Also, the methylmercury exposure due to consumption of contaminated rice seems to impair the levels of the intelligence quotient (IQ) [15]. These findings suggest a negative association between exposure to methylmercury and intelligence. Amidst this backdrop of environmental and sociocultural shifts, the Ninam Association from the State of Roraima (Texoli), acting on behalf of the Yanomami community, sought the aid of researchers to investigate the ramifications of mining operations in the Yanomami territory, with a specific focus on mercury exposure and its detrimental health impacts.

## 2. Materials and Methods

This is a cross-sectional study carried out between 4 and 14 October 2022 in the Yanomami Indigenous Territory, located in Alto Mucajaí, Roraima state. The total indigenous population of the Alto Mucajaí Region is 605 people, with 247 being under the age of 12. Indigenous residents of the region live in nine villages. Eight out of the nine invited villages were represented by children who attended the study. The research team set up camp in Aldeia Lasasi, where living conditions, energy supply, sanitation, and food provisions were inadequate. Indigenous individuals journeyed to the village by foot or boat, traversing considerable distances, as the location is nestled within the heart of the Amazon Rainforest, devoid of motor vehicles or road access. The entire population was invited to participate in the survey, and we obtained 120 participating children. Factors that hindered participation included the distances traveled by boats, weather conditions such as rain, as well as other adversities and social issues. The research group was made up of 17 health professionals and researchers who carried out clinical assessments and interviews using pre-structured questionnaires for data collection. The entire process was accompanied by indigenous interpreters with the purpose of translating from the Ninan/Yanomami language into Portuguese and vice versa.

In total, 120 children under 12 years of age were included in the study, with the consent of their parents or guardians. The variables studied were place of residence, fish consumption, nut consumption, school attendance, learning difficulties, family income, mercury levels in the hair, physical and anthropometric examination, hemoglobin level in the blood, and Total Intelligence Quotient (TIQ).

### 2.1. Ethical Aspects

This project was evaluated and approved by the research ethics committee of the National School of Public Health (Fundação Oswaldo Cruz). Before starting the interviews and evaluations, the study was explained to all leaders and residents of the invited villages. The leaders then agreed and signed the free and informed consent form.

### 2.2. Clinical and Pediatric Evaluation

The pediatric assessment consisted of analysis of the child’s health and vaccination booklets, anthropometric measurements, clinical examination, and capillary hemoglobin measurement. Two different examiners performed the anthropometric measurements, using a calibrated digital scale and a portable anthropometer and, for analysis, the average of the two measurements was considered. Weight and height values were classified into Z-Scores weight/age, height/age, and BMI/age according to the 2006 World Health Organization curves. Z-Score values, corresponding to age, below −2 were classified as low weight (weight and BMI) and short stature (height). The calculations were carried out on the WHO Anthro and WHO Anthro Plus platforms, developed and made available by the World Health Organization (WHO) [16]. For children up to 10 years old, weight/age, BMI/age and height/age Z-Score calculations were performed. For those over 10 years of age, Z-Score BMI/age and height/age were calculated.

The assessment of capillary hemoglobin dosage was carried out using portable hemoglobin analyzer HB 301-System (Hemocue^®^, Ängelholm, Sweden), eliminating the need to collect and store venous blood samples. Children were considered anemic when hemoglobin levels were below 11.5 g/dL for those over 6 years old, and below 11 g/dL for those under 6 years old [17].

### 2.3. Cognitive Evaluation

The cognitive assessment was carried out by a trained psychologist who applied the following tests: Snijders-Oomen Non-verbal Intelligence Test (SON-R, applied to children between 2 and a half years and 7 years and 11 months of age) and the Wechsler Abbreviated Scale of Intelligence (WASI, applied to children between 8 and 11 years and 11 months of age).

Prior to the evaluation, the instruments were presented to two indigenous teachers who were native speakers of Ninan and fluent in Portuguese. The teachers then assisted in the process of translating and applying the instruments, as well as making the necessary adaptations to the context of the indigenous population. The SON-R cognitive test did not undergo any changes as it is a non-verbal test. On the other hand, the WASI Scale, which has verbal components, was adapted with guidance from teachers.

### 2.4. Mercury Analysis

Hair samples were collected to evaluate the mercury levels, in order to reflect the levels of mercury that the individual was exposed to in the past month due to ingestion of contaminated fish. Briefly, samples were collected from the occipital region, stored in paper envelopes, and sent to the Toxicology Laboratory, in the Environment Section of the Evandro Chagas Institute (IEC), in Belém-Pará, Brazil, for a total mercury levels (THg) analysis. The full methodology is described elsewhere [18].

### 2.5. Statistical Analysis

For the statistical analysis, continuous variables (weight, height, BMI, hair mercury levels, hemoglobin levels, family income, and IQ) were initially subjected to the Shapiro–Wilk test, along with graphical analysis. Subsequently, they were represented with mean and standard deviation (SD) and analyzed using the Student’s *t*-test. Linear regression models were applied to investigate the relationship between continuous variables, and residuals were graphically analyzed. The variable used as the outcome in the statistical model was the Total Intelligence Quotient (TIQ), and the independent variables were hair mercury levels, age, fish consumption, and Brazilian nut consumption.

To enter continuous variables in the model that did not present a normal distribution, they were transformed into square roots to meet the assumption of linearity. Categorical variables were represented as number (N) and percentage (%) and analyzed using Pearson’s chi-square test. For all analyses, a significance level of 5% was considered. All statistical analyses were performed using the R Software (R Foundation for Statistical Computing, Vienna, Austria, version 4.2.2)

## 3. Results

A total of 120 children, ranging from newborns to 11 years and 11 months old, participated in the pediatric evaluation (average: 75.4 months, standard deviation: 39.4; minimum: 3.0; maximum: 143.9 months). During the assessments, 47 children (39.2%) were under 5 years of age, and among them, 13 (10.8% of the total) were under 2 years old. Children between 6 and 9 years old and those aged 10 to 11 years constituted 35.8% and 25.0% of the sample, respectively. There was a slight predominance of boys (52.5%) over girls (47.5%), with no statistically significant differences between age groups (Table 1).

Roughly one-third of caregivers reported that their child had been hospitalized at least once, with a slightly higher proportion of hospitalizations among girls (35.1%) compared to boys (27.0%) (*p*-value: 0.337) (Table 1). Approximately one-third of caregivers (n = 36; 30% of the total) stated not having their child’s health booklet. Among those who presented the health booklet for verification (n = 84), it was observed that only 15.5% of the children were up to date with vaccinations according to the national immunization schedule. Vaccine coverage was slightly higher among boys (19.0%) compared to girls (11.9%) (*p*-value: 0.365) (Table 1).

**Figure 1:**
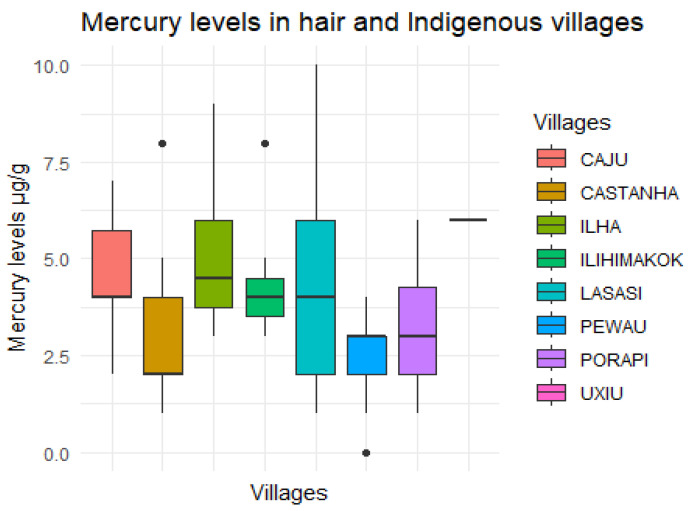
Hair mercury levels according to villages, Yanomami Indigenous Territory, Roraima State, Brazilian Amazon, 2022. Total Hg dosed from hair samples, missing information from 3 children. Dots represent outliers.

The Total Hair Mercury (Total Hg) levels in µg/g were assessed in 117 children and categorized as “<2.0 µg/g” and “≥2.0 µg/g” for both girls and boys. Among girls, 10 had a value below 2.0 µg/g, and 47 had a value above 2.0 µg/g. Among boys, 14 had a value below 2.0 µg/g, and 46 had a value above 2.0 µg/g. No significant difference was observed between genders (*p*-value = 0.438, Table 1). However, it was noted that 93 children (79.4%) had a value exceeding 2.0 µg/g. The Hg levels ranged from 0.16 µg/g to 10.20 µg/g, with a mean of 3.30 µg/g and a median of 3.70 µg/g. Regarding the place of residence and the level of mercury in the children’s hair, it was observed that the highest mercury levels were found in the Ilha, Lasasi, Ilihimakok, Castanha and Caju villages (Figure 1).

The average hemoglobin level among the tested children (n = 110) was 12.1 g/dL (standard deviation: 1.3; minimum: 7.6; maximum: 16.4 g/dL), and the prevalence of anemia reached 27.3%, with a slight predominance among girls (29.4%) compared to boys (25.4%) (*p*-value: 0.640) (Table 1). In the Lasasi, Caju, and Ilha villages, the highest prevalences were identified, with 38.2%, 33.3%, and 25.0% of the children having anemia, respectively.

For the assessment of the Total Intelligence Quotient (TIQ), 58 neurodevelopmental tests were administered, including 32 SON-R and 26 WASI. Among the 58 children for whom it was possible to estimate the Total Intelligence Quotient (TIQ), the average value was 68.6, ranging from 42 to 92 points (median: 69.5; standard deviation: 10.5; 25th percentile: 60.7 points; 50th percentile: 69.5 points; 75th percentile: 77 points). The typical ranges employed by the Wechsler scale are as follows: 90–109, considered average; 80–89, low average; 70–79, borderline; and <70, extremely low. These classifications were utilized in accordance with the test manual to categorize the sample [19]. The TIQ mean of the evaluated children was 68.6 and the median was 69.5, indicating that the intellectual functioning of these children was extremely low, according to the Wechsler classification. In this group, 55.2% of the evaluated children showed intelligence deficit; 34.5% were considered borderline; 8.6% had a lower median index. 

**Figure 2:**
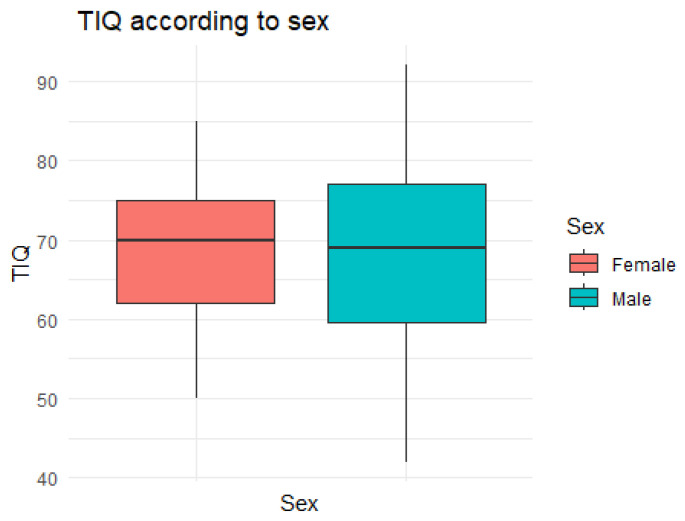
Total Intelligence Quotient (TIQ) values according to sex, Yanomami Indigenous Territory, Roraima State, Brazilian Amazon, 2022.

Figure 2 presents the values of the Total Intelligence Quotient (TIQ) test according to sex. There were no statistically significant differences between genders (*p*-value: 0.788; Table 1). Only one seven-year-old boy, residing in the Lasasi village, showed an intelligence index considered median for his age.

The majority of the children resided in the Lasasi village (51.3%), followed by the Porapi (21.1%), Pewaú (20.8%), and Caju (12.5%) villages (Table 2). Approximately two-thirds of the families of the assessed children reported receiving an income above US$120.00 (64.2%), 32.5% reported an income of up to US$120.00, and 3.3% claimed to have no income at all. There was no statistically significant difference between the families of girls and boys (*p*-value: 0.975) (Table 2). The majority of caregivers (63.3%) reported that their child regularly attends school in the village, with a slight predominance of girls (68.4%) compared to boys (58.7%). However, 15.8% of caregivers claimed that their child faces difficulties in staying in school, with the highest percentage of difficulties observed in boys (*p*-value: 0.445) (Table 2). Regarding dietary habits, 88.3% of caregivers reported that their children regularly consume fish, with a slight predominance among boys (92.1%) compared to girls (84.2%) (*p*-value: 0.118) (Table 2). Finally, 90.8% of caregivers reported that their children regularly consume nuts.

The analysis of the nutritional status (Table 3) indicates that the median of the weight-for-age indicator was −1.86 Z-scores (standard deviation: 0.9; minimum: −4.2; maximum: 0.19); the median of the height-for-age indicator was −2.66 Z-scores (standard deviation: 1.0; minimum: −5.7; maximum: −1.10); and the median of the BMI (Body Mass Index) was −0.17 Z-scores (standard deviation: 0.7; minimum: −4.17; maximum: 2.23). This reveals that 43.8% of the children have deficits in weight-for-age, and 77.3% have deficits in height-for-age.

To explore potential reasons for the low median Total Intelligence Quotient (TIQ) observed in the children, we conducted linear regression analyses to examine the relationships between independent variables and TIQ as the dependent variable. Several models were tested, and one was considered as best adjusted (Table 4). A negative association was found between the total IQ and the following variables: total mercury level, age, and fish consumption. Lower IQ scores were associated with higher mercury levels, older age, and fish consumption, adjusted for each other and by nut consumption (Table 4). The lowest score on the IQ test was associated with five times the risk of having high levels of mercury in their hair, two and a half times the risk of having an older age, and almost eight times the risk of consuming fish, adjusting for nut consumption.

## 4. Discussion

This study found that Yanomami children exhibit weight and height below the expected levels for their age. Furthermore, the investigation revealed heightened levels of mercury in the children’s hair, surpassing the safety threshold established by the World Health Organization (WHO) at 2.0 µg/g [20]. Notably, 93 children (79.4%) exceeded this threshold, raising concerns about potential elevated mercury exposure. The data also indicates a high prevalence of anemia and notably low IQ values.

Mercury poisoning, specifically from methylmercury (MeHg), can occur through contaminated food, primarily fish, consumed by the mother during pregnancy, through breastfeeding after birth, or through the consumption of fish in infant food. Fish consumption is prevalent among the indigenous child population, and there are beneficial nutrients present in fish and seafood that can counterbalance the adverse effects of low-level mercury exposure [21].

The neurotoxicity of mercury became known through studies of chronic exposure in pregnant women in Minamata Bay, Japan, due to water contamination, and in acute poisoning incidents in Iraq, caused by contaminated wheat [22]. Children born to asymptomatic pregnant women who were chronically exposed to high levels of methylmercury exhibited neurodevelopmental alterations such as motor and speech delays, sensory function impairment, severe intellectual disability, seizures, and, in some cases, coma and death [22,23]. Regarding chronic contamination of pregnant women and their children, some cohort studies assessed the effects of prenatal methylmercury exposure and observed alterations in child neurodevelopment, such as the Norway (Faroe Islands), Seychelles, New Zealand, and Japan cohorts. However, in Spain (Infancia y Medio Ambiente Cohort), the results did not show an association, and in the Avon Longitudinal Study of Parents and Children (ALSPAC) cohort, no impairment of neurodevelopment was found [22]. 

In a systematic review, researchers concluded that the evidence of the association between prenatal mercury exposure and neurodevelopmental alterations in children aged zero to five years is weak. However, the authors noted limitations to the study. The first is the use of different scales for neurodevelopmental assessment. The second concerns the difference between biomarkers and placental exposure. The third is the heterogeneity of the studies evaluated, and the fourth is the size and ability to detect minor developmental changes in children [24]. Transitioning from studies evaluating the effects of chronic mercury exposure to its neurotoxicity, the pathological pathway of methylmercury in mammalian neurogenesis is as follows: (1) alteration of cell proliferation, gene expression, and calcium homeostasis, (2) alteration in oxidative stress production, and (3) alteration in cell migration [25].

In a study carried out with Yanomami children evaluating their nutritional status, it was found that, in children under five years of age, the prevalence of low height to age index was 83.8% and low weight to age index was 50%, indicating a serious nutritional situation among the children [26]. Genetic, dietary, infectious, and environmental factors may be involved in the etiology of impaired growth among Yanomami children, in addition to the greater caloric expenditure involved in traveling in the forest [26]. In order to clarify some controversial points about this topic, a cohort study carried out among the Xavante indigenous people suggests that the growth pattern of these children, especially in the first six months of life, is comparable to the global reference median. However, from six to thirty-six months, this pattern becomes variable and below the reference median, possibly due to the influence of precarious sanitary conditions, food, and environmental insecurity on indigenous child growth [27]. 

Due to the inadequate living conditions prevalent in Brazilian indigenous communities, the height of parents may not reflect the genetic potential transmitted to their children; although genetic potential is a determining factor for the child’s final height, it is influenced by other factors [28]. For example, social inequalities play a crucial role in the nutrition and health of children in low- and middle-income countries, resulting in excess deaths and malnutrition, often due to irregular provision and low quality of health services [26]. These arguments seem to apply to the reality experienced by the Yanomami and, more broadly, to the situation of indigenous peoples in Brazil.

In contrast to the presented above, a study carried out in five Pataxó villages in Minas Gerais did not identify nutritional deficits among children. The majority of children lived in homes with electricity and basic sanitation. Of the pregnant women, 82.4% had six or more prenatal consultations and 91.2% started prenatal care in the first trimester. Recent hospital admissions were mainly for non-infectious causes and the vaccination coverage was high. The researchers concluded that better housing conditions, sanitation and child health care may have contributed to the good nutritional status of Pataxó children [29]. 

In the present study, we observed a delay in vaccination of mandatory vaccines under the National Immunization Program in 84% of children. Ensuring high vaccination coverage rates is essential to prevent the resurgence and spread of diseases, some of which have already been eliminated or eradicated in the country, in addition to preventing an increase in morbidity and mortality in several other conditions [30].

In the model that assessed total IQ, there was an association between low IQ levels and greater fish consumption, older age, and higher levels of mercury in the hair, which suggests that fish contaminated by MeHg may be causing damage to the neurodevelopment of children. Fish consumption is a source of omega-3 fatty acids, proteins, vitamin D, and other minerals essential for optimal fetal development [26]. However, studies indicate that fish intake during pregnancy may modify the relationship between prenatal meHg exposure and neurodevelopmental outcomes, suggesting that the beneficial effects of nutrients present in fish may lessen the adverse effects of exposure to low levels of Hg [31]. However, it is possible that older children consume greater amounts of fish, in addition to not using breastfeeding, which has many other nutrients and neuroprotective factors [32].

Santos-Lima et al. [11] evaluated 263 children between 6 and 14 years of age living in the Amazon region, from the riverside population of the Madeira River (Rondônia state), using several neuropsychological tests; among them, they evaluated the association between general intelligence and mercury levels in hair. This study suggests an association of higher mercury levels in children’s hair with worse performance in intelligence tests. Although this is a study with riverside children, an important part of the protein consumption of this community is fish. Similarly, our results suggest that poorer performance on the TIQ test is associated with higher fish consumption, older age in children, and elevated levels of mercury in hair, taking into account the ingestion of Brazilian nuts.

Regarding school attendance, most children are enrolled in a school with basic infrastructure and a limited teaching staff, in which teachers serve children of different age groups simultaneously, adopting the format of multi-grade classes. Still, considering school age is from 6 years old, school attendance is predominant. Learning difficulties were reported by 19 of the 70 children who attended the school, generally pointed out by parents or translators present at the time of the interview. Despite observing, in Table 1, that 32 out of 58 children who underwent IQ testing showed intellectual deficiency, it can be noted, from Table 2, that only 19 children reported learning difficulties. One assumption that could explain such a result would be the lack of perception about what the question entails, by the respondent who answered the survey. 

The local cultural context may focus on other tools of child neurodevelopment, such as hunting, fishing, household chores, and survival skills in the forest. It is a cultural difference in itself that, for indigenous people, such activities are more important than going to school. For these families, considering that the child is capable of performing complex activities such as hunting, domestic activities, and planting is more valued than school performance, and this is a cultural difference to be considered. It may also be for these reasons that respondents, in addition to not understanding the depth of the issue, do not give the same importance to school learning functions as other cultures value. 

The TIQ test results revealed a worrying average of 68.5. A value equal to or less than 70 on the WASI test is indicative of a possible diagnosis of intellectual disability [33]. The test result may demonstrate cultural and linguistic limitations, despite the translators present and the adaptation of some items to the local reality. Other elements to be considered as influences for intellectual disability include biological factors, such as malnutrition, and environmental factors, including exposure to violence and heavy metals [11,33]. Another determining factor for considering intellectual disability is adaptive behavior, which is a standard set of responses at a conceptual, social, and practical level that the child can perform when faced with situations in the environment in which they are inserted. It has to do with the efficiency with which the child takes care of himself and relates to others (DSM V) [34]. In this context, what an indigenous child can do in their social, practical, cultural context and the way they relate to others, is a skill that would require more comprehensive and accurate investigation for the Yanomami population [34].

Child malnutrition signals social inequalities, chronic diseases, deficits in executive functions, risk of early mortality, and potential reduction in income in adulthood [35]. Our results showed a high prevalence of short stature, low weight, and BMI very close to the limit for classifying malnutrition, in addition to children with low family income. The scenario where the indigenous child is exposed to various adverse conditions, accentuated by exposure to methylmercury, signals a serious situation that deserves the attention of competent bodies. High prevalence of anemia, which is reported to be higher in indigenous populations, can be explained by intestinal parasitic diseases that disrupt intestinal iron absorption, the presence of malaria, and low consumption of iron-rich foods [36]. Indigenous communities face several challenges, including inadequate living conditions, insufficient consumption of iron and other essential micronutrients in their diets, and high rates of infectious diseases [37]. Anemia is often related to nutritional deficiencies and health problems, and its higher prevalence among indigenous people reflects disparities in access to healthcare, food security, and living conditions in general [37]. In a study that evaluated the main factors related to anemia in children, it was shown that age, low maternal education, low socio-economic status, malnutrition, diarrhea, insufficient iron intake in the diet, and high consumption of cow milk are the most common factors associated with anemia [38].

The children had a familiar household income with an average equivalent to USD 255 per month (BRL 1278.00), which is 2.3 times lower than the average usual income of the Brazilian population during the same period as the study [39].

Despite the illustrative findings, this present study has some limitations, highlighting that, as it is a cross-sectional study, it is not possible to establish causal relationships or consider the temporality of the events. Another limitation lies in linguistic difficulties, which require the assistance of translators during psychological assessments. Furthermore, considerations about the specific cultural differences of indigenous populations are relevant, as they differ from the populations for which intelligence tests were originally developed. Even the non-verbal test (SON-R) applied to children between 2 and 7 years of age has an adaptation and validation that does not correspond to the indigenous reality. This constitutes another limitation of the study, as the standardized instruments used to assess children’s cognition do not match what can be expected in terms of a reality as diverse as the indigenous reality. This same difficulty is highlighted in other studies of indigenous peoples in Peru [12,13].

However, it should be noted that this area of research is explored through expeditions led by multidisciplinary teams, destined for places that are difficult to reach and contact. Therefore, carrying out longitudinal observations is more challenging, limiting their contribution to an area of research lacking data in the literature. At the same time, this was, to our knowledge, the first work to evaluate the TIQ scores and their relation to chronic mercury exposure in the Yanomami child population. On the other hand, it is equally important to consider the difficulties related to cultural differences and the non-standardization of instruments for this population, considering learning and potential of neurodevelopment in children. Biological, socio-economic, and environmental factors can negatively impact children’s neurodevelopment. The neurological development of these children over the years can be compromised, restricting the full expression of their capabilities in adulthood.

## 5. Conclusions

The present study observed a negative association between the lowest score on the TIQ test and increasing age, mercury levels, and fish consumption. This suggests that mercury residue from illegal mining on indigenous lands may have an adverse impact on the neurodevelopment of indigenous children, especially those who consume fish, despite the benefits of consuming the fish itself. Moreover, the nexus between biological, environmental, and social disparities is noteworthy, as indigenous children grapple with intertwined challenges that mutually influence one another, leading to adverse impacts on their well-being.

Nevertheless, it is crucial to acknowledge the obstacles linked to cultural disparities and the absence of standardized tools tailored for this demographic, while also considering the learning process and the neurodevelopmental potential in children. The disruption of neurodevelopment in children is influenced by various factors and could be compromised over time, influenced by additional variables that might act as mediators and interactors, aspects which were not explored within this study.

Additional research findings indicate weight and height indices below the general population average, compared to the non-indigenous population, along with a high prevalence of anemia and reduced levels of Total Intelligence Quotient (TIQ), indicating an unfavorable environmental and socio-economic condition. These findings highlight the urgency of improving the quality of health care, ensuring food security, improving basic sanitation, and implementing protection measures for the Indigenous Territory, always respecting the culture and way of life of traditional peoples.

## Figures and Tables

**Table 1:** Sociodemographic and clinical characteristics of the study population of the Yanomami Indigenous Territory, Roraima State, Brazilian Amazon, 2022 (n = 120).

Clinical Characteristics	Female		Male		Total		*p*-Value
Age (months)	n	%	n	%	n	%	0.375
0–6	2	3.5	0	0.0	2	1.7	
6–12	0	0.0	3	4.8	3	2.5	
12–24	4	7.0	4	6.3	8	6.7	
24–36	4	7.0	4	6.3	8	6.7	
36–60	10	17.5	16	25.4	26	21.7	
60–108	23	40.4	20	31.7	43	35.8	
108–144	14	24.6	16	25.4	30	25.0	
Total	57		63		120		
Hospitalization							0.337
No	37	64.9	46	73.0	83	69.2	
Yes	20	35.1	17	27.0	37	30.8	
Total	57		63		120		
Up-to-date vaccination ^1^							0.365
No	37	88.1	34	81.0	71	84.5	
Yes	5	11.9	8	19.0	13	15.5	
Total	42		42		84		
Anemia ^2^							0.64
No	36	70.6	44	74.6	80	72.7	
Yes	15	29.4	15	25.4	30	27.3	
Total	51		59		110		
Total Hg (µg/g) ^3^							0.438
<2.0 µg/g	10	17.5	14	23.3	24	20.5	
≥2.0 µg/g	47	82.5	46	76.7	93	79.5	
Total	57		60		117		
IQ Total ^4^							0.788
Intelligence deficit	15	55.6	17	54.8	32	55.2	
Borderline	10	37.0	10	32.2	20	34.5	
Low Average	2	7.4	3	9.7	5	8.6	
Average	0	0.0	1	3.2	1	1.7	
Total	27		31		58		

^1^ Vaccination according to the immunization schedule of the Brazilian Ministry of Health. ^2^ Hemoglobin levels lower than 11.5 g/dL for children over 6 years of age, and lower than 11 g/dL for children under 6 years old. ^3^ Total Hg dosed from hair samples, missing information from 3 children. ^4^ Total IQ obtained by the Snijders-Oomen Non-verbal Intelligence Test (SON-R, from 2 ½ to 7 years old) and the Wechsler Abbreviated Scale of Intelligence (WASI, from 8 to 11 years old) tests.

**Table 2:** Socio-economic characteristics of the study population of the Yanomami Indigenous Territory, Roraima State, Brazilian Amazon, 2022 (n = 120).

Sociodemographic Characteristics	Female		Male		Total		*p*-Value
Residence Village	n	%	n	%	n	%	0.909
Castanha	8	14.0	5	7.9	13	10.8	
Pewaú	11	19.3	14	22.2	25	20.8	
Cajú	6	10.5	9	14.3	15	12.5	
Ilha	2	3.5	2	3.2	4	3.3	
Ilhimakok	3	7.7	4	10.8	7	9.2	
Lasasi	18	46.2	21	56.8	39	51.3	
Porapi	8	20.5	8	21.6	16	21.1	
Uxiu	1	2.6	0	0.0	1	1.3	
Total	57	47.5	63	52.5	120		
Household Income							0.975
No income	2	3.5	2	3.2	4	3.3	
Income up to US $120	19	33.3	20	31.7	39	32.5	
Income above US $120	36	63.2	41	65.1	77	64.2	
Total	57		63		120		
School Attendance							0.333
Not applicable	1	1.8	4	6.3	5	4.2	
No	17	29.8	22	34.9	39	32.5	
Yes	39	68.4	37	58.7	76	63.3	
Total	57		63		120		
Learning difficulty							0.445
Not answered	18	31.6	27	42.9	45	37.5	
Do not know	3	5.3	2	3.2	5	4.2	
No	28	49.1	23	36.5	51	42.5	
Yes	8	14.0	11	17.5	19	15.8	
Total	57		63		120		
Fish consumption							0.118
No	9	15.8	5	7.9	14	11.7	
Yes	48	84.2	58	92.1	106	88.3	
Total	57		63		120		
Brazilian Nut consumption							0.623
No	6	10.5	5	7.9	11	9.2	
Yes	51	89.5	58	92.1	109	90.8	
Total	57		63		120		

**Table 3:** Anthropometrics characteristics of the study population of the Yanomami Indigenous Territory, Roraima State, Brazilian Amazon, 2022 (n = 120).

	Median (Min; Max)
Weight for Age ^1^	−1.86 (−4.21; 0.19)
Height for Age ^2^	−2.66 (−5.70; 1.10)
BMI for Age ^2^	−0.17 (−4.17; 2.23)

^1^ Z-scores of weight for age, for those children under 10 years old. ^2^ Z-scores of height for age and BMI for age, for all children.

**Table 4:** Results from the linear regression model between Total Intellectual Quotient and mercury levels, age, and consumption of fish and nuts.

Dependent Variable	Independent Variable	*β*	*p*-Value	R^2^	Normality of Residuals (*p*-Value)
Total IQ	Total Hg (µg/g)	−5.13	0.048	0.32	0.57
	Age	−2.52	0.001		
	Fish consumption	−7.8	0.040		
	Brazilian nut consumption	3.93	0.46		

## Data Availability

Data are contained within the article.
